# Neuroinflammation is linked to dementia risk in Parkinson’s disease

**DOI:** 10.1093/brain/awad322

**Published:** 2023-09-27

**Authors:** Antonina Kouli, Lennart R B Spindler, Tim D Fryer, Young T Hong, Maura Malpetti, Franklin I Aigbirhio, Simon R White, Marta Camacho, John T O’Brien, Caroline H Williams-Gray

**Affiliations:** Department of Clinical Neurosciences, University of Cambridge, Cambridge, CB2 0PY, UK; Department of Clinical Neurosciences, University of Cambridge, Cambridge, CB2 0PY, UK; Department of Clinical Neurosciences, University of Cambridge, Cambridge, CB2 0PY, UK; Wolfson Brain Imaging Centre, University of Cambridge, Cambridge, CB2 0QQ, UK; Department of Clinical Neurosciences, University of Cambridge, Cambridge, CB2 0PY, UK; Wolfson Brain Imaging Centre, University of Cambridge, Cambridge, CB2 0QQ, UK; Department of Clinical Neurosciences, University of Cambridge, Cambridge, CB2 0PY, UK; Department of Clinical Neurosciences, University of Cambridge, Cambridge, CB2 0PY, UK; Wolfson Brain Imaging Centre, University of Cambridge, Cambridge, CB2 0QQ, UK; Medical Research Council Biostatistics Unit, Cambridge Institute of Public Health, Cambridge, CB2 0SL, UK; Department of Clinical Neurosciences, University of Cambridge, Cambridge, CB2 0PY, UK; Department of Psychiatry, University of Cambridge, Cambridge, CB2 0SZ, UK; Department of Clinical Neurosciences, University of Cambridge, Cambridge, CB2 0PY, UK

**Keywords:** Parkinson’s disease, dementia, PET imaging, inflammation, tau

## Abstract

The development of dementia is a devastating aspect of Parkinson’s disease (PD), affecting nearly half of patients within 10 years post-diagnosis. For effective therapies to prevent and slow progression to PD dementia (PDD), the key mechanisms that determine why some people with PD develop early dementia, while others remain cognitively unaffected, need to be understood. Neuroinflammation and tau protein accumulation have been demonstrated in post-mortem PD brains, and in many other neurodegenerative disorders leading to dementia. However, whether these processes mediate dementia risk early on in the PD disease course is not established. To this end, we used PET neuroimaging with ^11^C-PK11195 to index neuroinflammation and ^18^F-AV-1451 for misfolded tau in early PD patients, stratified according to dementia risk in our ‘Neuroinflammation and Tau Accumulation in Parkinson’s Disease Dementia’ (NET-PDD) study. The NET-PDD study longitudinally assesses newly-diagnosed PD patients in two subgroups at low and high dementia risk (stratified based on pentagon copying, semantic fluency, *MAPT* genotype), with comparison to age- and sex-matched controls. Non-displaceable binding potential (BP_ND_) in 43 brain regions (Hammers’ parcellation) was compared between groups (pairwise *t*-tests), and associations between BP_ND_ of the tracers tested (linear-mixed-effect models). We hypothesized that people with higher dementia risk have greater inflammation and/or tau accumulation in advance of significant cognitive decline. We found significantly elevated neuroinflammation (^11^C-PK11195 BP_ND_) in multiple subcortical and restricted cortical regions in the high dementia risk group compared with controls, while in the low-risk group this was limited to two cortical areas. The high dementia risk group also showed significantly greater neuroinflammation than the low-risk group concentrated on subcortical and basal ganglia regions. Neuroinflammation in most of these regions was associated with worse cognitive performance (Addenbrooke’s Cognitive Examination-III score). Overall neuroinflammation burden also correlated with serum levels of pro-inflammatory cytokines. In contrast, increases in ^18^F-AV-1451 (tau) BP_ND_ in PD versus controls were restricted to subcortical regions where off-target binding is typically seen, with no relationship to cognition found. Whole-brain ^18^F-AV-1451 burden correlated with serum phosphorylated tau181 levels. Although there was minimal regional tau accumulation in PD, regional neuroinflammation and tau burden correlated in PD participants, with the strongest association in the high dementia risk group, suggesting possible co-localization of these pathologies. In conclusion, our findings suggest that significant regional neuroinflammation in early PD might underpin higher risk for PDD development, indicating neuroinflammation as a putative early modifiable aetiopathological disease factor to prevent or slow dementia development using immunomodulatory strategies.

## Teaser

About half of all people with Parkinson’s disease develop dementia within 10 years post-diagnosis. Kouli, Spindler *et al*. show using PET scans that patients with higher dementia risk have increased inflammation early on, suggesting that this could be a potential treatment target to prevent dementia.

## Introduction

Dementia is one of the most devastating aspects of Parkinson’s disease (PD), and treatment options at present remain extremely limited with no therapies available to prevent or slow its development. Our previous longitudinal study in a population-based incident PD cohort showed that 46% of patients develop dementia within 10 years from diagnosis,^1^ corroborating previous cumulative prevalence estimates of 83% at 20 years from diagnosis.^2^ It remains incompletely understood why some PD patients develop early dementia while others remain cognitively intact throughout their illness, and what factors determine the marked variability in rates of cognitive decline. While extensive research has determined that age, male sex and various predisposing genotypes are associated with greater risk of developing PD dementia (PDD),^3,4^ these risk factors are not modifiable through therapeutic intervention.

Two key processes that are implicated in various neurodegenerative dementias and may contribute to cognitive decline in PD are tau accumulation and neuroinflammation. Both of these are plausibly therapeutically modifiable aetiopathological factors for PDD. However, antibody and alternative strategies for tau clearance are not yet available—whereas many anti-inflammatory treatments are already licensed for other conditions and thus readily available for drug repurposing studies. Our neuropathological studies have demonstrated evidence of heightened neuroinflammation in cognition-relevant brain regions in post-mortem brain tissue from PDD cases compared to cognitively intact PD.^5^ Alzheimer’s type changes, including neurofibrillary tau tangles, are also found in a significant proportion of PDD cases post-mortem.^6,7^ Furthermore, genome-wide association studies have provided strong evidence that the tau gene *MAPT* (microtubule-associated protein tau) locus is associated with PD,^8,9^ and homozygosity for a common inversion polymorphism in *MAPT* (H1 haplotype) has been linked to dementia risk in PD,^1,10–15^ although this finding is not universally replicated.^16^ Analysis of post-mortem PD brain indicates that the H1/H1 genotype is associated with increased tau expression,^10,17^ thus providing a potential mechanistic link for the reported genetic association with PDD risk.

Both neuroinflammation and tau accumulation can be quantified *in vivo* with the use of PET radiotracers. To evaluate neuroinflammation, the principal radiotracer used over the past three decades has been ^11^C-PK11195: this radioligand binds to the mitochondrial translocator protein (TSPO), which is upregulated in activated microglia. In 2005, Ouchi *et al*.^18^ were the first to show increased inflammation in the midbrain of 10 newly-diagnosed PD patients compared to 10 matched controls. A number of studies have since demonstrated further increased ^11^C-PK11195 binding in later-stage PD, with studies including more advanced cases reporting *in vivo* neuroinflammation across multiple cortical and subcortical regions.^19^ Second generation TSPO ligands have also provided some supportive evidence for increased neuroinflammation in PD, although findings have been more variable across studies, with only increased binding in the midbrain identified as a robust finding in meta-analysis.^19^ However, recently, Yacoubian *et al*.^20^ showed in a larger cohort of newly-diagnosed, untreated PD patients that binding of the second generation TSPO ligand DPA-714 is significantly increased in various subcortical and cortical regions.

Studies evaluating TSPO binding in cognitively impaired PD cases have been limited, but Edison *et al*.^21^ investigated ^11^C-PK11195 binding in PDD alongside non-demented PD cases and reported increases in temporal, parietal and occipital cortices in both groups compared with controls, with additional involvement of frontal, temporal and cingulate regions in PDD subjects. Furthermore, increased ^11^C-PK11195 binding in PDD patients was associated with greater impairments in cognitive function, measured by the Mini-Mental State Examination (MMSE) score—thus providing *in vivo* evidence that brain inflammation may be involved in dementia progression in PD. Nevertheless, it remains unclear whether neuroinflammation is an epiphenomenon linked to accumulating neurodegenerative pathology or whether it actually precedes and contributes to the development of PDD.

For the *in vivo* measurement of misfolded tau protein, several tracers have been developed to date, with ^18^F-AV-1451 (also known as ^18^F-flortaucipir) being the only one approved by the US Food and Drug Administration following extensive use since its development in 2013.^22–24^^18^F-AV-1451 PET scanning in nine PD patients with mild cognitive impairment (PD-MCI) and 17 PD patients without cognitive impairment revealed no differences in the standardized uptake value ratio (SUVR) between the two groups or compared with controls.^25^ In contrast, another group investigated the retention pattern of ^18^F-Flortaucipir in patients with PD, dementia with Lewy bodies (DLB) and a mixed group of PD-MCI and PDD and observed a significant increase in SUVR in the inferior and lateral temporal lobe of cognitively impaired PD patients compared with controls, although to a lesser extent than for DLB.^26^ When combining DLB and cognitively impaired PD groups, greater ^18^F-AV-1451 uptake in the inferior temporal gyrus was strongly associated with worse MMSE scores. Similarly, significantly increased ^18^F-AV-1451 uptake in the medial and lateral parietal cortex of PDD compared with PD patients has also been reported to correlate negatively with letter S and animal fluency, though no associations were found with global cognition (MMSE).^27^

Taken together, PET imaging studies have provided evidence of *in vivo* neuroinflammation and tau accumulation in PD. However, it is unclear how these processes contribute to the earliest stages of dementia development in PD in advance of its clinical expression. This is critical to establish, given that the optimal window for therapeutic intervention is likely to be at, or before, the onset of the dementing process. Furthermore, no studies have explored how tau pathology and neuroinflammation may be related to one another *in vivo* in the context of cognitive dysfunction in PD. This study aimed to fill these central knowledge gaps by investigating neuroinflammation, brain tau accumulation and dementia risk in 36 newly-diagnosed PD cases and 20 age-matched controls using PET neuroimaging in a 3-year longitudinal study (NEuroinflammation and Tau aggregation in Parkinson’s Disease Dementia, NET-PDD). The experimental paradigm involves stratification of PD cases at the point of diagnosis according to their risk of developing an early dementia. This is based on predictors determined from our long-term population-based incident PD cohort study, CamPaIGN (Cambridgeshire Parkinson’s Incidence from GP to Neurologist), namely, impaired pentagon copying, semantic fluency performance and the *MAPT* H1/H1 genotype,^1,10,11,28^ with the latter chosen as a genetic predictor with particular relevance given our aim to investigate tau accumulation. Here, we present the baseline neuroinflammation and tau PET data from the NET-PDD study. Binding potential—a metric of binding site density—for ^11^C-PK11195 and ^18^F-AV-1451 was compared across a comprehensive map of brain regions in these newly-diagnosed PD ‘high dementia risk’ and ‘low dementia risk’ cases compared with age- and gender-matched controls.

We hypothesized that (i) patients in the high dementia risk PD group would have the highest levels of brain inflammation and/or tau accumulation at this early point in the disease course; (ii) the degree of neuroinflammation would correlate with tau burden in the brain; and (iii) regional neuroinflammation and/or tau accumulation would be associated with early deficits in cognitive function. We also explored correlations between brain inflammation and tau accumulation, as well as peripheral markers of these processes, which may constitute more accessible and readily-scalable biomarkers.

## Materials and methods

### Participants

Recently diagnosed PD cases (≤2 years disease duration, Hoehn and Yahr ≤2) fulfilling UK PD Brain Bank Criteria for diagnosis were recruited from the Parkinson’s Disease Research Clinic at the John Van Geest Centre for Brain Repair, University of Cambridge. Patients with PD were stratified into dementia risk subgroups based on predictors previously identified from the CamPaIGN longitudinal cohort study.^1,10,11,28^ Existing *MAPT* genotypic data and clinical data from baseline research clinic assessments were used for stratification. Discrimination between *MAPT* H1 and H2 haplotypes had previously been performed by genotyping for the rs9468 single nucleotide polymorphism using a TaqMan allelic discrimination assay (ID: C_7563752_10; Thermo Fisher Scientific #4351379). Genotyping was done on a QuantStudio 12 K Flex Real-Time PCR System (Applied Biosystems). Patients within either high or low dementia risk groups were recruited to the study. Patients in the high dementia risk group had the H1/H1 *MAPT* genotype and a semantic fluency score < 20 or impaired pentagon copying. Patients in the low dementia risk group were carriers of the H2 *MAPT* allele and had a semantic fluency score ≥ 20 and unimpaired pentagon copying. Exclusion criteria for PD patients were a diagnosis of dementia according to the Movement Disorder Society (MDS) PD-dementia criteria^29^ and significant psychiatric disturbance. Age, sex and *MAPT* genotype-matched controls with no history of neurological disease or depression were recruited from the National Institute for Health and Care Research Cambridge Bioresource (http://www.cambridgebioresource.org.uk) or from the Parkinson’s Disease Research Clinic. Exclusion criteria for both patient and control groups were the presence of chronic inflammatory or autoimmune disorders, current or latent infection, vaccinations in the preceding month and use of anti-inflammatory/immune-modulating medications, as well as suspected/manifest dementia based on the Addenbrooke’s Cognitive Examination-III (ACE-III) score. Participants attended for three visits, including a clinical/neuropsychological assessment, a ^11^C-PK11195 PET-MR scan and a ^18^F-AV-1451 PET-MR scan, with a 4.7 ± 2.8 month mean interval between the first and last visits. Ethical approval was obtained from the East of England—Essex Research Ethics Committee (16/EE/0445), and the study was approved by the UK Administration of Radioactive Substances Advisory Committee (ARSAC).

### Clinical and neuropsychological assessment

All participants were clinically assessed for comorbid conditions and medication history. PD cases underwent standardized assessments of motor and cognitive dysfunction, including the MDS-Unified Parkinson’s Disease Rating Scale (MDS-UPDRS, completed in the ON medication state), ACE-III and Beck Depression Inventory (BDI). Levodopa equivalent daily doses were calculated.^30^ Control participants were examined by a neurologist to confirm the absence of PD or other neurological disease and assessed using the MDS-UPDRS part III (motor examination), ACE-III and BDI.

### PET and MRI data acquisition and image reconstruction

All PET scans were conducted on a GE SIGNA 3 T PET/MR scanner (GE Healthcare). Immediately prior to PET tracer injection, an MR attenuation correction sequence (2-point Dixon; LAVA-Flex) was acquired to provide information utilized for attenuation correction of the PET data. List-mode PET data were acquired for 75 and 90 min following injection with ^11^C-PK11195 (405 ± 78 MBq) and ^18^F-AV-1451 (186 ± 10 MBq), respectively. The PET data were histogrammed into 55 and 58 time frames for ^11^C-PK11195 and ^18^F-AV-1451, respectively, and then reconstructed into images (128 × 128 × 89 matrix; 2.0 × 2.0 × 2.8 mm voxel size) using a time-of-flight version of ordered subsets expectation maximization,^31^ with 16 subsets, six iterations and no smoothing. Attenuation correction used a pseudo-CT generated by a multi-subject atlas method^32^ from a T1-weighted BRAVO MR image acquired during PET data acquisition (192 × 512 × 512 matrix interpolated to a 192 × 280 × 280 matrix with 1.0 mm isotropic voxel size), together with an improved MRI head coil attenuation template.^33^ Image reconstruction also included corrections for random coincidences, dead time, normalization, scattered coincidences, radioactive decay and sensitivity.

### PET data analysis

SPM12 (https://www.fil.ion.ucl.ac.uk/spm/software/spm12/) was used to realign each dynamic image series and co-register each realigned dynamic PET image series to the BRAVO MR image from the same scan. To estimate specific tracer binding, both ^11^C-PK11195 and ^18^F-AV-1451 PET data were analysed with the simplified reference tissue model (SRTM)^34^ to quantify binding potential relative to a non-displaceable compartment (BP_ND_). For ^11^C-PK11195, the reference region was estimated with supervised cluster analysis, with correction for vascular binding included in the model.^35^ For ^18^F-AV-1451, the grey matter probability map estimated by SPM12 from the BRAVO MR was first smoothed to PET spatial resolution, and then a 90% lower threshold was applied to produce a grey matter reference region in the inferior portion of the cerebellum. For regional analysis, an adapted version of the n30r83 Hammersmith atlas (http://brain-development.org) was transformed to each BRAVO MR using ANTs (https://picsl.upenn.edu). This atlas includes various regions of interest (ROIs) that are established as important in PD histopathological studies (e.g. substantia nigra and putamen). An additional whole brain grey matter ROI was defined by applying a 50% lower threshold to the SPM12 grey matter probability map smoothed to PET spatial resolution. The time-activity curve of each ROI was corrected for CSF contamination through division with the mean sum of grey and white matter probabilities in the ROI, with both probability maps smoothed to PET spatial resolution. SRTM was then applied to the CSF-corrected ROI time-activity curves.

### Measurement of inflammatory markers and tau in serum

Venous blood samples were collected in S-Monovette tubes at the clinical assessment visit. The samples were allowed to clot for 15 min prior to centrifugation at 2000 rpm for 15 min. Serum was removed and stored in 200 mcl aliquots at −8°C until assays were performed. A panel of key inflammation-related markers was measured using Meso Scale Discovery (Rockville) S-PLEX electrochemiluminescent immunoassays for IFN-γ, IL-1β, IL-6, TNF-α and IL-17. These were selected based on markers shown to be associated with PD disease status and/or more rapid disease progression in the literature.^36–38^ Serum phosphorylated (p)-tau181 (a marker shown to be elevated in the plasma of PD patients versus controls^39^) was measured using a Quanterix SIMOA Advantage V2 SR-X kit. Assays were run according to the manufacturer’s instructions, and all samples were processed in duplicate.

### Statistical analysis

For each tracer, statistical analysis used BP_ND_ values from three central ROIs (midbrain, pons and medulla), together with average values for 40 bilateral ROIs, thereby allowing a detailed analysis of the tracer binding spatial distribution. Comparisons of ^11^C-PK11195 and ^18^F-AV-1451 BP_ND_ between the three groups used both frequentist and Bayesian Student’s *t*-tests. As frequentist tests of the null hypothesis cannot conclude in favour of the H_0_ but merely to its rejection, we also used Bayesian statistics to enable inferences to be made with confidence on the absence of correlation and/or prediction. Traditional concepts of type I and type II errors do not apply to Bayesian statistics, where one instead determines the relative model evidence in favour of the null (H_0_) or in favour of the alternate hypothesis (H_1_), or that the evidence is indeterminate from the data available–thus forgoing the need to correct for multiple comparisons. The parameter BF_10_ (known as the Bayes factor) that we report is a measure of the evidence in favour of H_1_ over H_0_. For instance, a BF_10_ = 8 means that there is eight times as much evidence for H_1_ than for H_0_. A BF_10_ between 1 and 3 is regarded as ‘mild’, between 3 and 10 as ‘moderate’, between 10 and 30 as ‘strong’ and above 30 as ‘very strong’ evidence for H_1_ over H_0_. Such an approach is particularly useful when small sub-samples are compared, as it reduces the danger of false positives and negatives common to purely frequentist analyses.

The relationship between ^11^C-PK11195 and ^18^F-AV-1451 BP_ND_ values was assessed using a linear mixed effects model with ^11^C-PK11195 BP_ND_ as independent variable and ^18^F-AV-1451 BP_ND_ as dependent variable. Random effects of brain region and person were included in the model. The relationships between either ^11^C-PK11195 or ^18^F-AV-1451 regional BP_ND_, clinical measures and serum markers were assessed using Spearman correlation. A frequentist *P*-value <0.05 was defined as statistically significant. R software (R Core Team, 2012) with ggplot toolboxes (CRAN, 2023) and the software package JASP (v.16.0.0.0) were used for statistical analyses.

## Results

### Demographics

A total of 36 PD patients and 20 age- and sex-matched controls were recruited. Three PD patients were unable to tolerate any scanning due to neck pain, and two were excluded due to subsequent diagnoses of glioblastoma and multiple system atrophy, respectively. Given the exclusion criterion of suspected dementia, one control participant was excluded due to an ACE-III score of 69 on neuropsychological assessment. Hence *n* = 31 PD patients (*n* = 15 low dementia risk, *n* = 16 high dementia risk) and *n* = 19 controls were included in the analyses. The demographic and clinical characteristics of the participants are shown in Table 1.

**Table 1:** Demographic and clinical characteristics of Parkinson’s disease and control groups

–	Control	PD	*P-*value	PD low dementia risk	PD high dementia risk	*P-*value
Sample size	19	31	–	15	16	–
Sex, % male	58%	63%	0.886	60%	75%	0.67
Age at first visit, years	65.6 (7.6) [55–77]	67.3 (7.4) [52–79]	0.277	66.7 (7.8) [54–79]	67.8 (7.6) [52–79]	0.68
Disease duration, years (time from diagnosis to study recruitment/first visit)	–	1.0 (0.6) [0.3–2.3]	–	1.3 (0.7) [0.3–2.3]	0.8 (0.4) [0.3–1.6]	0.015*
ACE-III total	96.7 (2.7) [88–100]	92.2 (4.8) [82–100]	<0.001***	94.0 (4.3) [85–100]	90.4 (4.7) [82–96]	0.041*
Pentagon copying	2.0 (0)	1.97 (0.18)	0.364	2.0 (0)	1.9 (0.3)	0.329
Semantic fluency score, 90 s	27.8 (4.6) [19–36]	25.0 (9.7) [13–51]	0.820	32.3 (8.5) [24–51]	18.1 (4.0) [13–30]	<0.001***
MDS-UPDRS motor	1.05 (1.5) [0–5]	28.4 (10.6) [10–46]	0.001***	24.4 (9.7) [10–43]	32.2 (10.3) [10–46]	0.045*
MDS-UPDRS total	–	47.7 (16.0) [22–81]	–	40.9 (14.9) [22–64]	54.0 (16.9) [23–81]	0.024*
Levodopa-equivalent daily dose, mg	–	271 (160)	–	248 (134)	293 (183)	0.55
BDI	4.1 (5.7) [0–24]	6.0 (4.9) [0–19]	0.228	5.5 (5.5) [0–19]	6.4 (4.4) [0–15]	0.641

Continuous variables were compared using *t*-test or Mann–Whitney U-test (for parametric and non-parametric data, respectively). Categorical variables were compared using chi-square test. Data are presented as mean ± standard deviation (SD) [range]. ACE-III = Addenbrooke’s Cognitive Examination III; BDI = Beck Depression Inventory; MDS-UPDRS = Movement Disorder Society-Unified Parkinson’s Disease Rating Scale; PD = Parkinson’s disease.

*P < 0.05, ***P < 0.001.

No significant differences in age, sex and levodopa-equivalent daily dose were found between PD high and low dementia risk groups. As anticipated, total ACE-III scores were lower in the PD group compared with controls (*P* < 0.001) and lower in the PD high dementia risk group than in the low dementia risk group (*P* = 0.041). Disease duration (defined as time from diagnosis to study recruitment/first visit) was slightly shorter in the PD high dementia risk group (*P* = 0.015) than in the low dementia risk group, and MDS-UPDRS III scores were higher in the high versus low dementia risk PD groups (*P* = 0.024).

### ^11^C-PK11195 PET

**Figure 1:**
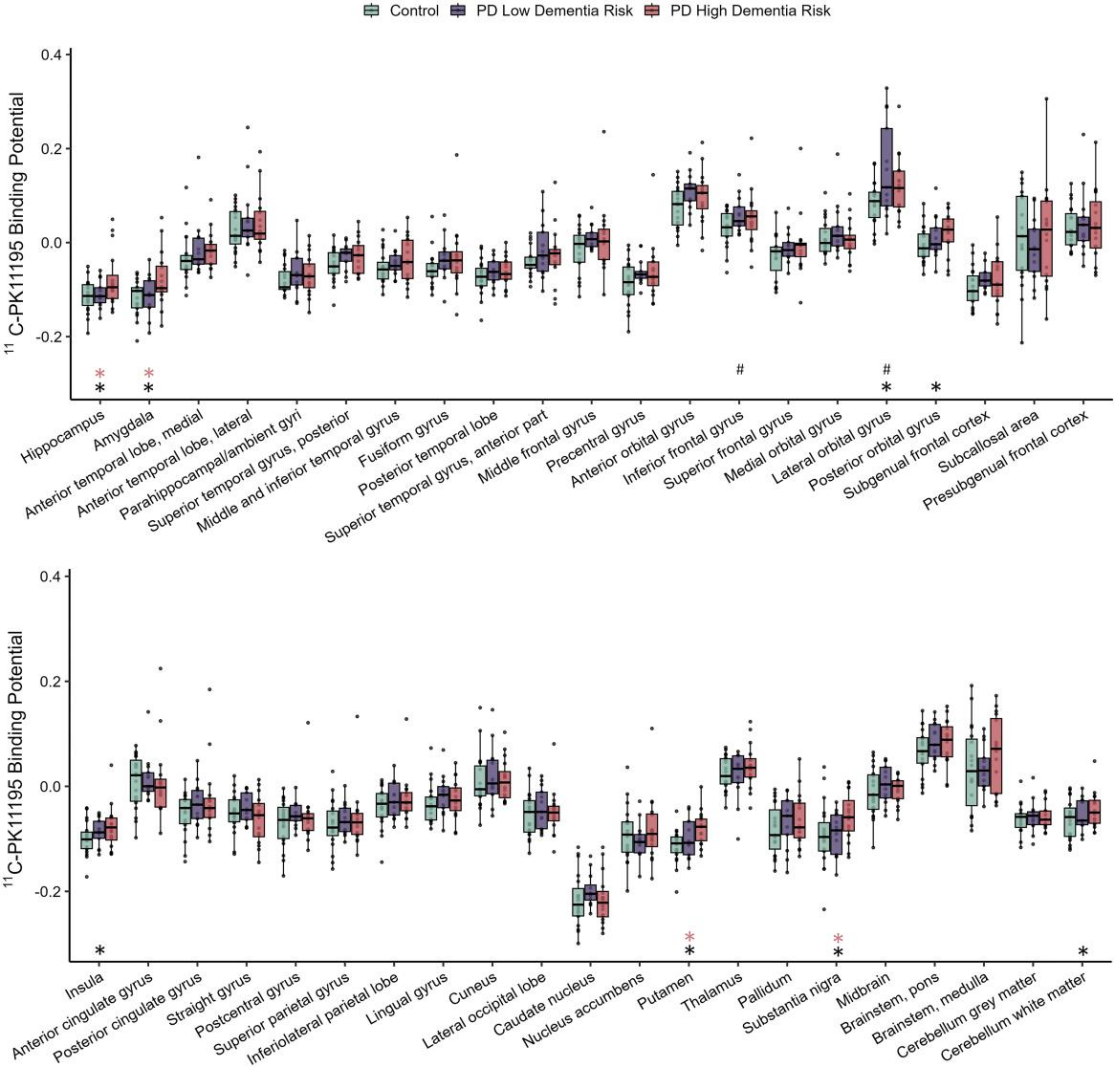
**^11^C-PK11195 non-displaceable binding potential in Parkinson’s disease patients at low and high risk of dementia.** Tukey box plots of ^11^C-PK11195 binding potential in controls, low dementia risk, and high dementia risk patients with Parkinson’s disease (PD). The box plots show individual data-points, the median and the interquartile range. *Significant difference between PD high dementia risk patients and controls. ^#^Significant difference between PD low dementia risk patients and controls. Red asterisks indicate significant difference between PD high dementia risk and PD low dementia risk groups. Tukey whiskers demarcate furthest values that lie within 1.5 interquartile ranges.

**Figure 2:**
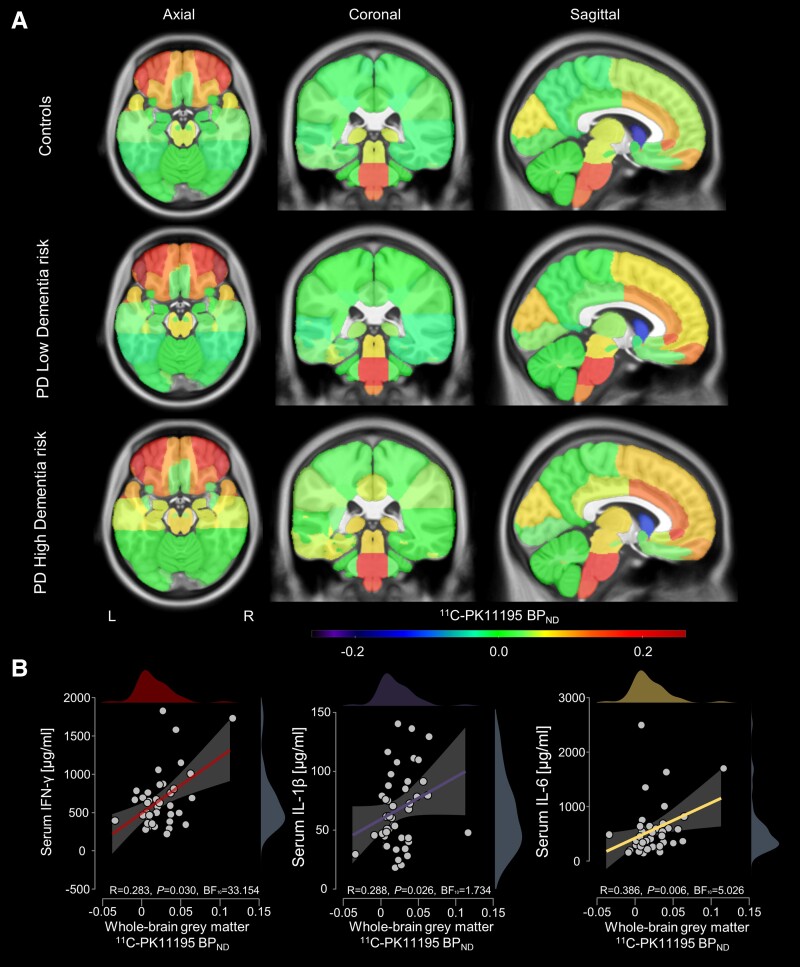
**Regional mean ^11^C-PK11195 non-displaceable binding potentials overlaid on ICBM152 template and correlations with serum cytokines.** (**A**) Parkinson’s disease (PD) patients with high dementia risk showed various increased regional ^11^C-PK11195 non-displaceable binding potential (BP_ND_) values compared with controls; in amygdala, hippocampus, putamen and substantia nigra, increases were statistically significant in PD high dementia risk compared to PD low dementia risk patients. (**B**) Whole-brain PK11195 BP_ND_ correlated with serum levels of three of the five pro-inflammatory cytokines assessed in both controls and PD participants (see Supplementary Fig. 2 for whole brain PK11195 BP_ND_ for all cytokines and subgroup analysis of PD only). BF_10_ = Bayes factor.

A total of 19 controls, 13 PD low dementia risk (two exclusions: one due to neck pain/not tolerating the scan, and one due to an unsuccessful tracer injection) and 16 PD high dementia risk participants underwent successful ^11^C-PK11195 scanning. To identify whether high dementia risk in PD may be associated with greater neuroinflammation, we compared regional ^11^C-PK11195 BP_ND_ in the PD patient groups against controls. In concordance with our hypothesis, widespread increased ^11^C-PK11195 BP_ND_ was observed in the PD high dementia risk group when compared with controls, with significantly increased BP_ND_ in the hippocampus (*P* = 0.016, BF_10_ = 4.20), amygdala (*P* = 0.012, BF_10_ = 5.22), putamen (*P* = 0.002, BF_10_ = 21.8), substantia nigra (*P* = 0.030, BF_10_ = 2.58), posterior orbital gyrus (*P* = 0.034, BF_10_ = 2.36), lateral orbital gyrus (*P* = 0.039, BF_10_ = 2.15), insula (*P* = 0.047, BF_10_ = 1.89) and the dentate cerebellum (*P* = 0.016, BF_10_ = 4.10; Figs 1 and 2A, Table 2 andSupplementary Fig. 1).

**Table 2:** Brain regions with significantly elevated ^11^C-PK11195 non-displaceable binding potential in Parkinson’s disease high and low dementia risk patients compared with controls

Region of interest	*P-*value | BF_10_
**PD high dementia risk group versus controls**	
Hippocampus	0.016 | 4.12
Amygdala	0.012 | 5.22
Putamen	0.002 | 21.88
Substantia nigra	0.030 | 2.58
Insula	0.047 | 1.89
Cerebellum dentate	0.016 | 4.10
Lateral orbital gyrus	0.039 | 2.15
Posterior orbital gyrus	0.034 | 2.36
**PD low dementia risk group versus controls**	
Lateral orbital gyrus	0.007 | 7.86
Inferior frontal gyrus	0.031 | 2.58
**PD high dementia risk group versus PD low dementia risk group**	
Hippocampus	0.047 | 1.97
Amygdala	0.041 | 2.15
Substantia nigra	0.028 | 2.82
Putamen	0.048 | 1.92

BF_10_ = Bayes factor; PD = Parkinson’s disease.

In contrast, in the PD low dementia risk group, elevated ^11^C-PK11195 BP_ND_ (compared with controls) was present only in the lateral orbital gyrus (*P* = 0.013; BF_10_ = 7.86) and inferior frontal gyrus (*P* = 0.034, BF_10_ = 2.58).

Comparison of ^11^C-PK11195 BP_ND_ between the PD high dementia risk group and the PD low dementia risk group showed significantly greater ^11^C-PK11195 BP_ND_ in the substantia nigra (*P* = 0.028; BF_10_ = 2.82), putamen (*P* = 0.048, BF_10_ = 1.92), amygdala (*P* = 0.035; BF10 = 2.15) and hippocampus (Welch’s test due to non-normality, *P* = 0.038, BF_10_ = 1.97).

To assess whether there was any relationship between neuroinflammation and peripheral inflammation, we explored correlations between whole-brain aggregate ^11^C-PK11195 BP_ND_ levels and a panel of five pro-inflammatory cytokines assessed in serum taken from both controls and PD patients (Fig. 2B). Significant correlations were observed for IFN-γ (R = 0.283, *P* = 0.030, BF_10_ = 33.154), IL-1β (R = 0.288, *P* = 0.026, BF_10_ = 1.734) and IL-6 (R = 0.368, *P* = 0.006, BF_10_ = 5.026). Correlations with TNF-α (R = −0.006, *P* = 0.517, BF_10_ = 0.174) and IL-17 (R = 0.062, *P* = 0.342, BF_10_ = 0.261) were non-significant (Supplementary Fig. 2). The correlations with IL-6, IFN-γ and IL-1β remained significant when analysis was restricted to PD patients (shown in Supplementary Fig. 2). Mean [± standard deviation (SD)] concentrations of inflammatory cytokines in controls, and high and low dementia risk PD groups, are shown in Supplementary Table 1. No significant between-group differences in cytokine levels were observed either between PD participants and controls or between the high and low risk group.

### ^18^F-AV-1451 PET

**Figure 3:**
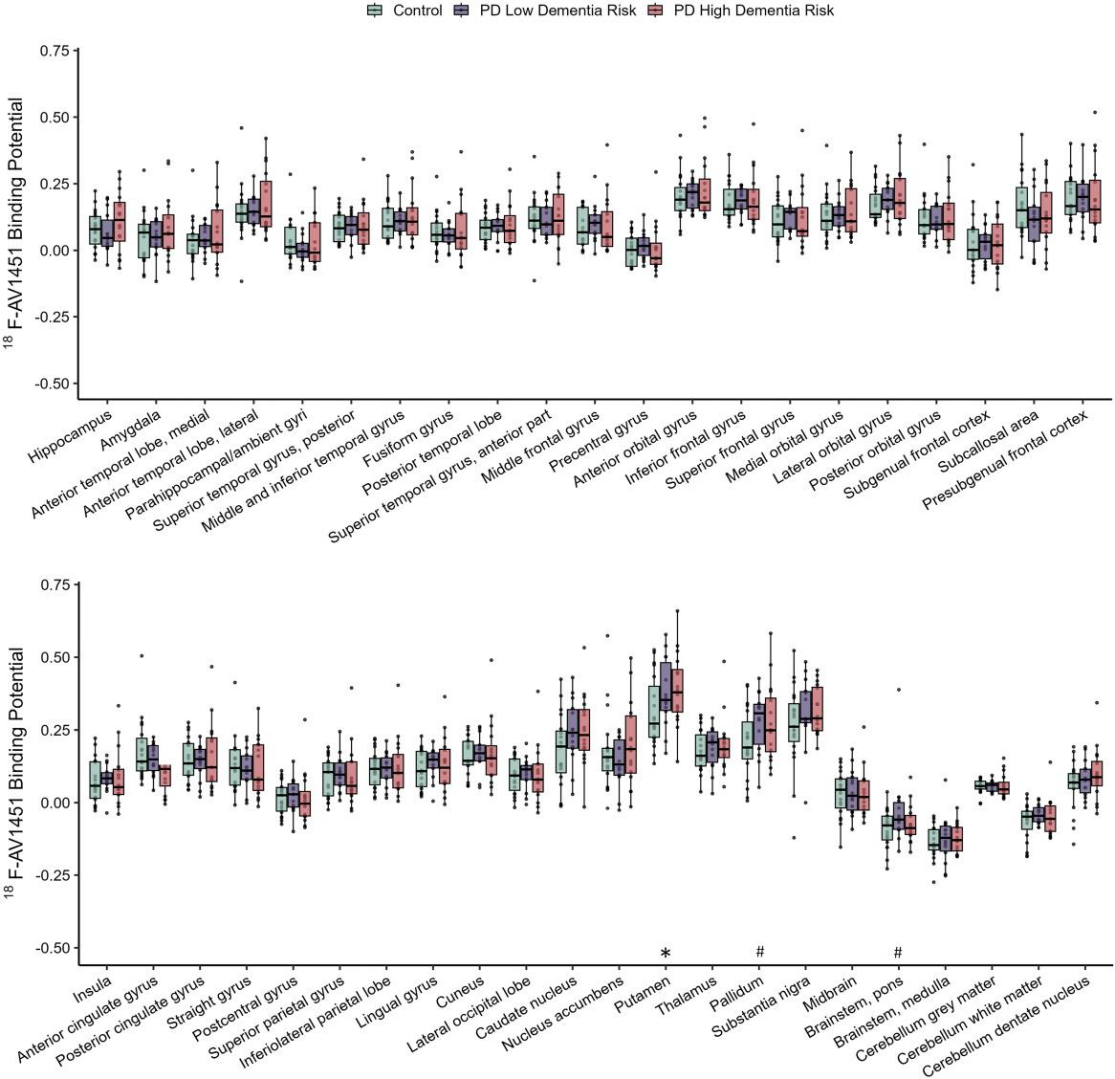
**^18^F-Flortaucipir binding potential (BP_ND_) in controls, PD low dementia risk, and PD high dementia risk patients.** The boxplots show the median and interquartile range. *Significant difference between PD high dementia risk patients and controls. ^#^Significant difference between PD low dementia risk patients and controls. Tukey whiskers demarcate furthest values that lie within 1.5 interquartile ranges.

**Figure 4:**
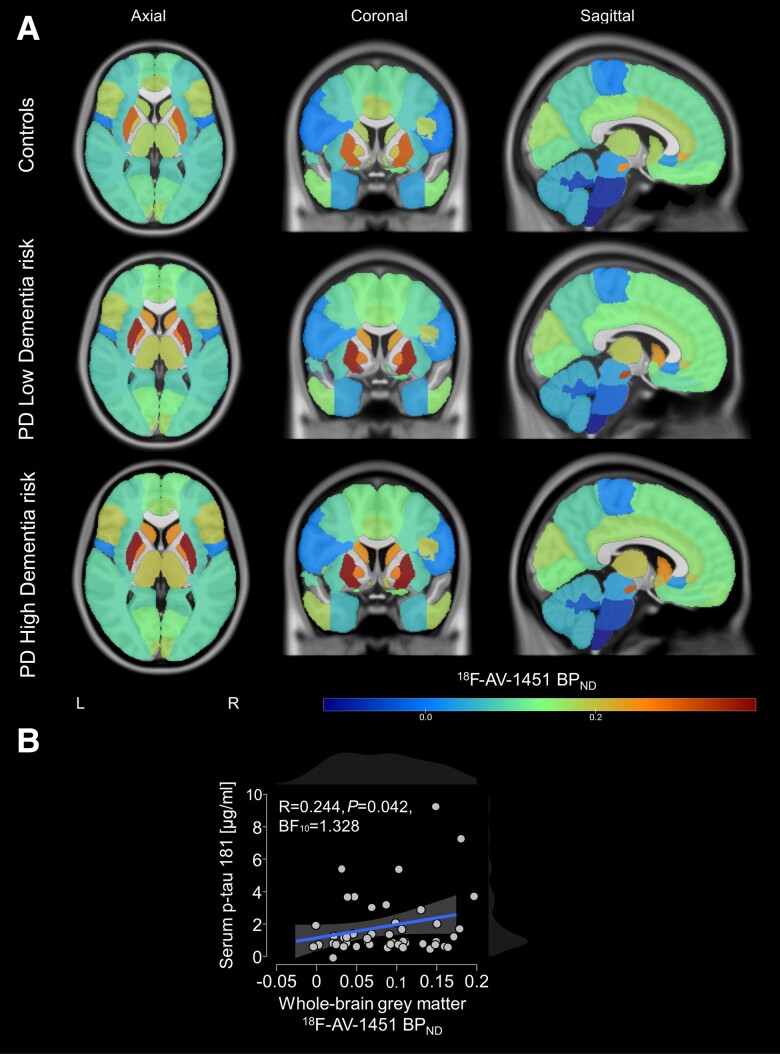
**Regional mean ^18^F-AV-1451 non-displaceable binding potentials overlaid on ICBM152 template and correlation with serum phosphorylated tau181.** (**A**) There were no significant differences outside of regions with established off-target binding. (**B**) Whole-brain AV-1451 non-displaceable binding potentials (BP_ND_) showed a significant association with serum phosphorylated (p)-tau181 levels. BF_10_ = Bayes factor.

A total of 19 controls, 15 PD low dementia risk and 16 PD high dementia risk participants underwent successful ^18^F-AV-1451 scanning. Analysis of regional ^18^F-AV-1451 BP_ND_ showed only very restricted elevations for both PD dementia risk groups in comparison with controls (see Figs 3 and 4A for brain plots). In high dementia risk PD patients, greater ^18^F-AV-1451 BP_ND_ was observed only in the putamen (*P* = 0.030, BF_10_ = 3.542), while in low dementia risk PD patients, increases were found only in the pallidum (*P* = 0.023, BF_10_ = 3.140) and pons (*P* = 0.044, BF_10_ = 1.975; Fig. 3 and Supplementary Fig. 2). ^18^F-AV-1451 binding in these brainstem and striatal regions was likely to represent ‘off target’ binding of the tracer to epitopes other than neurofibrillary tau tangles as widely reported in the literature.^40–42^ Specifically, the greater binding in the PD participants may have been due to age- and possibly pathology-related neuromelanin and iron deposits, for which AV-1451 shows binding affinity.^41,43^ There were no significant differences observed between the high and low dementia risk PD groups.

We also explored the relationship between whole brain aggregate ^18^F-AV-1451 binding and serum levels of p-tau181, finding a significant correlation (R = 0.244, *P* = 0.042; Fig. 4B). Mean serum p-tau181 levels are shown in Supplementary Table 1. There were no significant differences between PD participants and controls or between the different dementia risk subgroups.

### Relationship between regional ^11^C-PK11195 and ^18^F-AV-1451 BP_ND_

**Figure 5:**
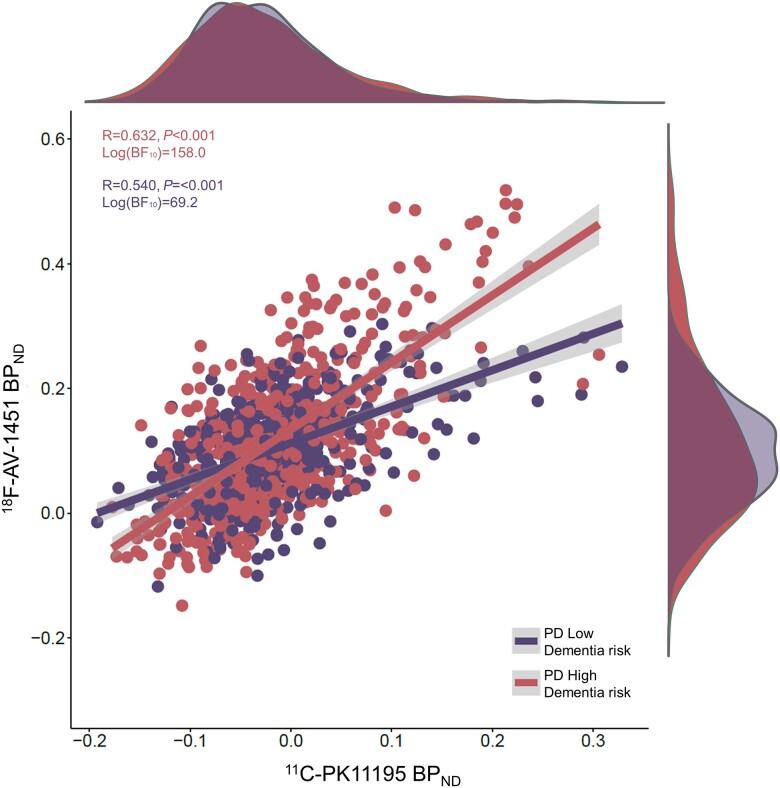
**Regional correlation between ^11^C-PK11195 BP_ND_ and ^18^F-Flortaucipir BP_ND_.** The dots represent individual regional values for each participant in the PD low dementia risk group and the PD high dementia risk group. ^11^C-PK11195 BP_ND_ showed a significant effect on ^18^F-Flortaucipir BP_ND_ at the whole cohort level in a linear mixed effects model, but only in the PD high dementia risk group was a significant correlation observed. BF_10_ = Bayes factor.

Whilst we observed marked significant regional increases in PK11195 binding but not AV-1451 binding, we wanted to explore whether tracer binding levels may be associated with one another to investigate whether both pathological processes may co-localize at this early time point in the PD disease course. We excluded all regions established or suspected to be off-target binding regions for AV-1451 (all basal ganglia, brainstem and cerebellum). Using a linear mixed effects model approach, we found a significant association between regional ^18^F-AV-1451 BP_ND_ and ^11^C-PK11195 BP_ND_ [random effect of participant and ROI; estimate = 0.866, standard error (SE) = 0.028, *t* = 30.803, *P* < 0.001] across the whole study population (all patients and controls who underwent successful scanning with both PET tracers; *n* = 29 PD participants and *n* = 19 controls). When considering the dementia risk subgroups, a significant correlation coefficient was observed for both the PD high dementia risk group [*n* = 15; R = 0.632, *P* < 0.001, log(BF_10_) = 158.0] as well as the PD low dementia risk group [*n* = 14; R = 0.540, *P* < 0.001, log(BF_10_) = 69.2]. However, the slope of the association between ^11^C-PK11195 and ^18^F-AV-1451 BP_ND_ was significantly greater in the high risk group (Fig. 5), evidenced by a significant interaction between group and ligand binding in an analysis of covariance of PD patient BP_ND_ values [*F*(1,26) = 16.771, *P* < 0.001, BF_10_ = 264.23]. These findings suggest that regional neuroinflammation may be associated with low grade tau accumulation, even though this is not sufficient to manifest in between-group differences in mean ^18^F-AV-1451 binding.

### Relationship between ^11^C-PK11195 BP_ND_ and clinical measures

We found a significant negative correlation between cognition (as measured by total ACE-III scores) and ^11^C-PK11195 BP_ND_ in several brain regions for the whole study population as shown in Table 3 (Spearman’s rank-order correlation, controlling for age at first visit). A linear regression with ACE-III as the dependent variable, whole brain grey matter ^11^C-PK11195 BP_ND_ as predictor and fixed effects of group and age as covariates showed a significant negative relationship only in the PD high dementia risk group (estimate = −4.944, SE = 1.328, *t* = −3.723, *P* < 0.001; BF_10_ = 2.811; low dementia risk PD group estimate = −1.866, SE = 1.408, *t* = −1.325, *P* = 0.192; BF_10_ = 0.149). No significant relationships between ^11^C-PK11195 BP_ND_ and either motor or total MDS-UPDRS scores were found across the whole participant group (controlling for age and levodopa equivalent daily dose), indicating that inflammation levels early-on in the disease course may relate more strongly to cognitive impairment than other PD-associated symptomatology.

**Table 3:** Spearman’s rank-order correlation between ACE-III and ^11^C-PK11195 non-displaceable binding potential in the total study population (*n* = 51)

Region of interest	ACE-III	
	Rho	*P-*value | BF_10_
Amygdala	−0.364	0.006 | 15.67
Posterior orbital gyrus	−0.312	0.025 | 4.47
Lateral orbital gyrus	−0.267	0.034 | 2.70
Putamen	−0.205	0.049 | 1.50
Substantia nigra	−0.251	0.045 | 1.46
Brainstem pons	−0.245	0.048 | 1.77
Brainstem medulla	−0.317	0.015 | 3.66
Cerebellum dentate	−0.396	0.003 | 24.70

Age as covariate. Rho is Spearman’s partial correlation coefficient (controlling for age; not corrected for multiple comparisons). ACE-III = Addenbrooke’s Cognitive Examination III; BF_10_ = Bayes factor.

### Relationship between ^18^F-AV-1451 BP_ND_ and clinical measures

There was no significant relationship between ^18^F-AV-1451 BP_ND_ and ACE-III scores in any brain region in the total study population or control/PD subgroups (Spearman’s rank-order correlation), nor were any correlations between ^18^F-AV-1451 BP_ND_ and MDS-UPDRS total and motor scores observed.

## Discussion

This study investigated the contribution of neuroinflammation and tau pathology to dementia risk in PD using a novel approach of stratifying newly-diagnosed patients based on their dementia risk. We provide evidence that recently diagnosed PD patients with a higher risk of developing dementia have increased neuroinflammation as measured by ^11^C-PK11195 binding in widespread subcortical (hippocampus, amygdala, insula, putamen, substantia nigra, cerebellum) and also some cortical (posterior and lateral orbital gyri) regions compared with age-matched controls, whereas in PD patients at low dementia risk, brain inflammation is much more regionally restricted. The level of PET-measured regional neuroinflammation/microglial activation correlates with pathological tau accumulation in the brains of PD patients and most strongly in those at higher dementia risk. Furthermore, levels of neuroinflammation in multiple cognition-relevant brain regions were correlated with cognitive impairment, whilst whole brain grey matter inflammation (overall inflammatory burden) was significantly associated with lower cognitive performance only in the PD high dementia risk group. In contrast, changes in tau accumulation (as measured by ^18^F-AV-1451 binding) were minimal in both PD subgroups compared with controls and did not correlate with cognitive impairment or other clinical measures of disease severity. Taken together, these findings demonstrated that neuroinflammatory changes occur early on in the PD time course and are linked to dementia risk—which supports the hypothesis that inflammation may be an early aetiopathogenic, potentially modifiable disease factor for PDD.

The majority of previous PET neuroimaging studies investigating neuroinflammation or tau in PD-associated cognitive impairment focused on cases in whom MCI or dementia were already diagnostically established. However, both of these pathological processes might play a key role at an earlier stage in the evolution of cognitive impairment. This motivated us to investigate the pathophysiological basis of the earliest stages of the dementing process in PD by comparing newly-diagnosed PD cases with either high or low risk of developing an early dementia. Our findings of widespread increased neuroinflammation in early PD cases at high dementia risk are in line with our previous work on inflammatory blood markers in PD: in newly diagnosed PD cases, proinflammatory markers were elevated to varying extents, with a more pro-inflammatory profile being predictive of faster disease progression and greater cognitive impairment.^36^ Furthermore, we have shown that inflammatory monocyte subsets are elevated in PD patients at higher dementia risk.^44^ The present results with ^11^C-PK11195 PET corroborate and extend these previous results by clarifying that brain inflammation is an early feature in those patients who are likely to develop a dementia phenotype. Furthermore, we have shown that brain inflammation in PD as measured by ^11^C-PK11195 binding is associated with peripheral inflammation (serum levels of IFN-γ, IL-1β and IL-6). This provides additional validation of ^11^C-PK11195 as a marker of inflammation, and adds weight to the hypothesis that peripheral and central immune activations are closely linked in PD. Importantly, the present study presents the baseline of an ongoing longitudinal study, which tracks cognitive and clinical progression over time and includes repeat ^11^C-PK11195 and ^18^F-AV-1451 PET imaging and peripheral biomarker analysis after 3 years. The resultant data will allow us to confirm whether baseline neuroinflammation and/or tau accumulation can serve as a predictive biomarker to forecast long-term cognitive outcomes and whether these markers may have utility to track disease progression, as well as effects of potential therapeutic interventions over time.

Whilst relatively few TSPO-PET studies have focused on cognitive impairment in PD, there is an expanding body of PET data acquired using both ^11^C-PK11195 and second generation TSPO ligands in PD compared with controls. Findings have been pooled in a recent meta-analysis, including ^11^C-PK11195 data from 116 PD cases and 95 controls; this demonstrated elevated binding across multiple cortical regions,^19^ partially in keeping with our findings in our high dementia risk PD group. The meta-analysis also showed increased binding in midbrain, substantia nigra, basal ganglia and thalamus regions, which are key sites of neurodegenerative pathology in PD. Some of these areas did reach significance in our analyses (e.g. substantia nigra, putamen, thalamus), but the midbrain areas did not. This may be due to differences in ROI definitions and parcellations, as well as patient populations, with several previous studies including only patients with advanced disease, whereas our cases were newly-diagnosed and early-on in the disease course. Indeed, a ^11^C-PK11195 study with a patient population similar to the one in our study did not find any binding increases in *de novo* PD cases (disease duration < 1 year) while more advanced cases had increased binding in the midbrain.^45^ Nevertheless, high dementia risk PD patients had the highest mean binding across all midbrain regions, with variability in binding possibly masking a difference between participant subgroups. It is also worthy to note that the dentate cerebellum showed elevated ^11^C-PK11195 binding in our study. While not often reported in PD cases, in atypical parkinsonism such as progressive supranuclear palsy, cerebellar inflammation has been observed in multiple analyses.^46^

Studies using second generation TSPO ligands have generally found less widespread neuroinflammation than those using ^11^C-PK11195, with a meta-analysis of second generation TSPO ligand data from 122 PD cases and 103 controls demonstrating increased binding in the midbrain for PD compared with controls but not in any of the other brain regions investigated.^14^ Two studies by the same group using ^18^F-FEPPA PET reported no significant differences between PD patients and controls,^47,48^ and similarly no differences between PD and controls were reported using ^11^C-PBR28.^49^ In contrast, longitudinal PET scanning with ^11^C-DPA-714 in early-stage PD patients (mean disease duration of 3 years) revealed a significant increase in binding in the temporal, parietal and occipital cortices, with a further increase primarily in the temporal and occipital cortex at 1-year follow-up.^50^ Furthermore, recent work by Yacoubian *et al*.^20^ demonstrated in a larger cohort of *de novo*, untreated PD patients that ^18^F-DPA-714 PET signal is significantly elevated in brain regions that overlap with those we found to have significantly elevated PK11195 binding.

Some of the disparities in findings from studies using first versus second generation TSPO tracers may relate to important differences in their binding characteristics as well as the kinetic modelling methodology used. Second generation tracers were developed to improve the signal-to-noise ratio, which is suboptimal with ^11^C-PK11195. However, unlike for ^11^C-PK11195, there is considerable heterogeneity of binding affinity for second generation tracers, which is genetically determined, with individuals displaying high, low or mixed affinity binding depending on a polymorphism (rs6971) in the *TSPO* gene.^51,52^ Patient stratification according to genotype is necessary in studies using these tracers, with exclusion of low affinity binders, which could introduce bias as the relevance and function of the TSPO polymorphism is currently poorly understood. It has also been suggested that PK11195 binding may not be distinguishable between low and high affinity binders due to lower signal-to-noise ratios than second generation ligands. However, the study by Yacoubian *et al*.^20^ shows similar dynamic ranges of DPA-714 to ^11^C-PK11195. In addition, most of the studies using ^11^C-PK11195 estimated neuroinflammation using BP_ND_ from reference tissue modelling, while most of the second generation TSPO ligand studies instead estimated total distribution volume (V_T_) from arterial input modelling; V_T_ in theory is a less specific metric of ligand binding, although it has the advantage of obviating the additional assumptions made with reference tissue modelling. It is important to note that we have followed the convention of many publications in referring to ^11^C-PK11195 binding as synonymous with neuroinflammation, despite the ligand having been designed as a measure of microglial activation and/or density. As TSPO upregulation may also occur in astrocytes, we here used the term neuroinflammation to capture the breadth of potential contributors to the ^11^C-PK11195 signal.

In the present study, we found no clear evidence to support the hypothesis that tau accumulation considered in isolation is a critical driver of the early development of PDD, with increased ^18^F-AV-1451 binding in the high and low dementia risk PD groups being found only in subcortical regions that are established sites of off-target binding with this particular tracer. Previous studies have reported similar increases in tracer uptake in the basal ganglia in elderly subjects, which likely reflects non-specific binding and correlates with iron deposition.^40,41^ Off-target binding to neuromelanin and iron is another limitation complicating the interpretation of ^18^F-AV-1451 binding, particularly in subcortical areas such as the substantia nigra. Off-target effects are less likely to be of relevance to cortical ^18^F-AV-1451 uptake, which has been reported to be increased in PDD and DLB patients. Smith *et al*.^27^ found increased ^18^F-AV-1451 SUVR in the medial and lateral parietal lobes of six DLB patients compared with 11 PD non-dementia patients and 44 controls as well as increased uptake in the medial parietal lobes of 18 PDD patients compared with the PD non-dementia group. The authors also found a significant negative correlation between verbal fluency scores and ^18^F-AV-1451 SUVR in the same parietal regions.^27^ Similarly, a recent study showed that longitudinal increases in ^18^F-AV-1451 SUVR in occipital, fusiform and inferior parietal cortices in DLB were associated with cortical atrophy and cognitive decline.^53^ Gomperts and colleagues^26^ investigated the retention pattern of ^18^F-AV-1451 in patients with PD, DLB and a mixed group of PD-MCI and PDD and observed a significant increase in SUVR in the inferior and lateral temporal lobe of DLB and PD-cognitively impaired patients compared with controls. When combining the DLB and PD-cognitively impaired groups, ^18^F-AV-1451 SUVR in the inferior temporal gyrus was found to correlate significantly with MMSE scores. These data implicate tau accumulation as a contributor to the progression of more advanced stage Lewy body dementias. However, our data indicate that in early pre-dementia PD cases, these binding patterns and associations with cognitive phenotype are not found, which highlights the possibility that neuroinflammatory changes may precede tau proteinopathy as a pathogenetic factor for cognitive decline in PD. Our findings that p-tau181 levels are associated with whole-brain ^18^F-AV-1451 load extend findings to the early PD context that have been made similarly in LBD with the tau-PET ligand ^18^F-RO948 and plasma p-tau181,^54^ and using CSF p-tau181 and the ligand 18F-MK6240 in a bigger cohort of healthy participants and individuals on the Alzheimer’s disease spectrum.^55^

To our knowledge, this is the first PET study in PD patients to use both ^11^C-PK11195 and ^18^F-AV-1451 in the same population. Although ^18^F-AV-1451 binding was not regionally elevated at the group level in PD cases at high dementia risk, we found significant regional correlations between binding of the two tracers with the greatest strength of association in the PD high dementia risk group. This relationship has been explored in the context of other neurodegenerative disorders with similar findings. Mak *et al*.^56^ found a significant association between the two tracers in four patients with LBD. A study by Malpetti *et al*.^46^ showed significant whole brain and regional correlation between ^11^C-PK11195 and ^18^F-AV-1451 BP_ND_ across 15 patients with progressive supranuclear palsy. Bevan-Jones *et al*.^57^ also found a strong correlation between the regional group means of the two tracers in patients with frontotemporal dementia (behavioural variant) and primary progressive aphasia (non-fluent and semantic variant). Hence our findings, taken together with previous studies, suggest that there is a relationship between ^11^C-PK11195 and ^18^F-AV-1451 binding both in primary tauopathies and alpha-synucleinopathies. This provides important *in vivo* evidence that brain inflammation and protein aggregation may co-localize early in the disease course, which corroborates and extends considerable *in vitro* and *in vivo* findings of associations between pathological protein aggregates and microglial activation. Indeed, recent evidence indicates that inflammation, and in particular activation of the inflammasome, may promote protein aggregation.^58^ Our results support this theory: whilst elevated regional brain inflammation is regionally linked to low grade tau accumulation, regional neuroinflammation is more prominent than tau accumulation in early disease compared with controls—which may suggest that inflammatory changes could precede significant tau deposition in the evolution of PDD. It is important to acknowledge that information about the amyloid status of the PD participants in this study was not available, and it is possible that amyloid co-pathology could at least partially drive inflammatory effects. The presence of amyloid co-pathology may contribute to the significant increases in inflammation detected with ^11^C-PK11195 PET in the cortical regions we found, which have shown early increases in amyloid pathology in DLB and Alzheimer’s disease.^59,60^ As such, the present results do not preclude that tau (and other protein) pathology contributes to the disease process but rather suggest that that inflammatory changes may be one of the most relevant early modifiable treatment targets in PD and other disorders.

The theory that neuroinflammation is relevant to the emergence of early cognitive dysfunction in PD is further supported by our finding of significant correlation between neuroinflammation and cognitive performance, particularly in the high dementia risk PD group. In contrast, we found no association with motor scores. Similarly, Yacoubian *et al*.^20^ recently also reported correlations between ^18^F-DPA-714 and cognitive, but not motor, measures in early PD. We found no significant relationship between ^18^F-AV-1451 binding and clinical measures. It is important to acknowledge that the exploration of such correlations is confounded by the narrow dynamic range in the clinical data of PD participants so early in the disease course, particularly the cognitive scores measured by ACE-III, which reduced power. This is a necessary limitation, given our goal to study the earliest stages of the dementing process, which necessitated recruitment of newly diagnosed patients with relatively homogeneous clinical/cognitive characteristics. Nevertheless, there were between group differences indicating increased disease severity in the high dementia risk group even though disease duration was actually shorter in this group—but this was not unexpected, given that high risk patients are on a more rapid disease trajectory. Furthermore, related to the strict inclusion criteria, our sample size was relatively small, although in line with other studies of PET ligands in PD and atypical parkinsonism disorders.^19^ Further limitations of our study relate to the ligands employed. ^11^C-PK11195 has a suboptimal signal-to-background noise ratio and low blood–brain barrier penetration.^61^ However, the use of second-generation TSPO tracers (with higher signal-to-noise ratio)^62^ is complicated by their genetically determined variability in binding affinity to TSPO,^51,52^ which adversely affects recruitment and statistical power, and potentially introduces bias in the population studied. In contrast, *in vitro* autoradiography studies indicated that ^11^C-PK11195 has a similar binding affinity across the population,^63^ and this was therefore the ligand of choice for the present study. Similarly, as already highlighted, an important caveat associated with the use of ^18^F-AV-1451 is its reported ‘off-target’ binding to neuromelanin,^64^ iron deposits^32^ and monoamine oxidase proteins,^42^ particularly in the basal ganglia. However, we took this into consideration and have reported this as likely to represent non-specific binding. Finally, our stratification of dementia risk only included the *MAPT* haplotype as a genetic risk factor, although recent studies have suggested that mutations in the gene *GBA* encoding glucocerebrosidase are the numerically greatest dementia risk contributors.^65^ We have *a posteriori* genotyped our participants and found that the all *GBA* mutation carriers (*n* = 5) in this PD cohort, aside from one, were classified as high dementia risk by our stratification method.

In conclusion, our results provide novel evidence that widespread brain inflammation is prominent in newly diagnosed PD cases who are at higher risk of early dementia. This suggests that therapeutic targeting of neuroinflammation as a modifiable disease factor in stratified PD cases who are at high dementia risk deserves further consideration. Longitudinal follow-up of our cohort will extend these findings on neuroinflammation, tau and their linkage, and establish the value of relevant PET markers in predicting long term outcomes and tracking disease progression over time.

## Supplementary Material

awad322_Supplementary_Data

## Data Availability

PET imaging and behavioural data are available upon request to the authors by qualified researchers. Requests will be considered on a case-by-case basis, assessing the feasibility and appropriateness of the proposed study and the capacity to maintain the required levels of data security, consistent with the original approved Research Ethics documentation and the patient information sheet that was the basis of consent obtained.
