# Supplementary material for: Neuroinflammation is linked to dementia risk in Parkinson’s disease
Source: Brain. 2023 Sep 27;147(3):923–35. doi: 10.1093/brain/awad322 (PMC10907093; doi:10.1093/brain/awad322)
Supplement: awad322_Supplementary_Data [file awad322_supplementary_data.pdf]

Supplementary Material

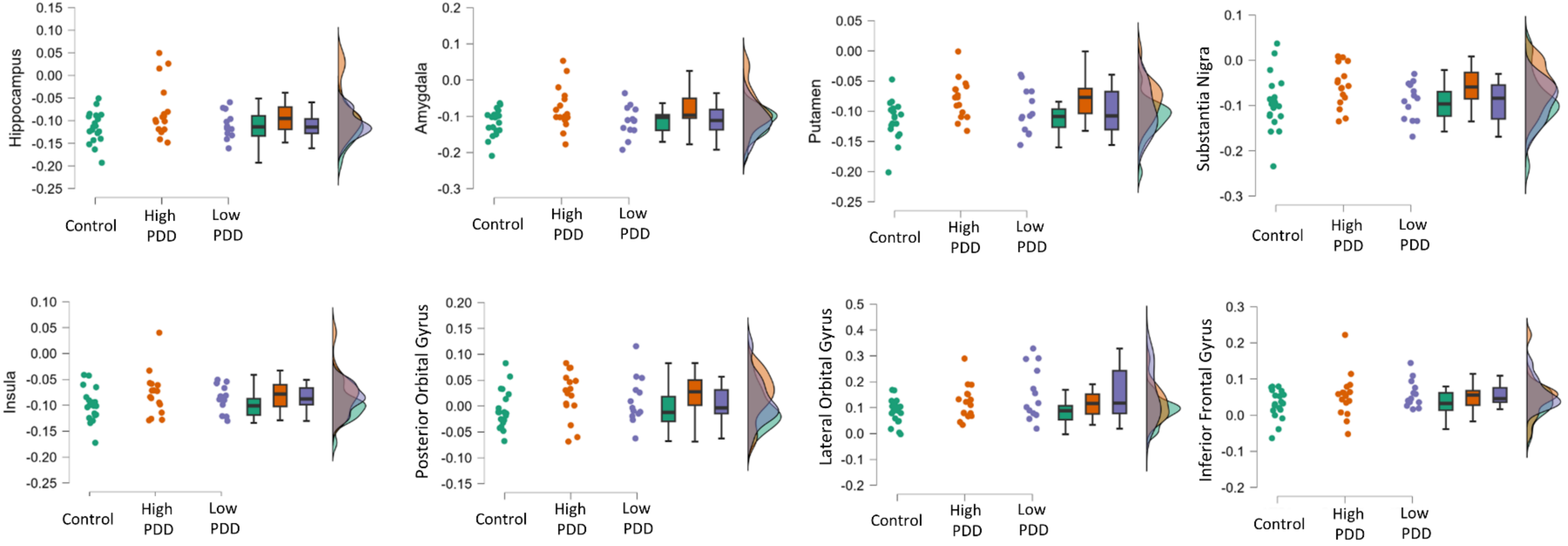

Supplementary Figure 1: Density raincloud plots of [^{11}C]PK11195 BP_{ND} in regions that showed significantly greater binding, either when comparing PD patients to controls or when comparing the PD low vs high dementia risk groups. Compare Table 3 for significance values and significant comparisons.

Table SM1. Serum biomarkers detected with MSD S-Plex or SIMOA assays. Raw values plus standard deviation and p-values from t-tests are shown.

	Control	PD	*p* value	PD low dementia risk	PD high dementia risk	*p* value
IL-1β	55.6 (±28.6)	72.4 (±32.1)	0.101	80.6 (±32.7)	65.7 (±30.9)	0.217
IFN-γ	667.2 (±373.0)	610.4 (±374.0)	0.560	577.0 (±364.8)	639.1 (±393.1)	0.682
IL-6	410.0 (±179.6)	638.793 (±549.9)	0.088	630.1 (±458.5)	645.3 (±624.5)	0.944
TNF-α	190.7 (±59.8)	198.75 (±98.2)	0.750	206.1 (±108.4)	192.8 (±92.5)	0.726
IL-17A	312.9 (±146.9)	350.6 (±239.4)	0.560	404.2 (±254.3)	307.0 (±225.2)	0.285
P-tau 181	1.764 (±1.6)	1.906 (±2.0)	0.797	1.746 (±2.3)	2.055 (±1.9)	0.689

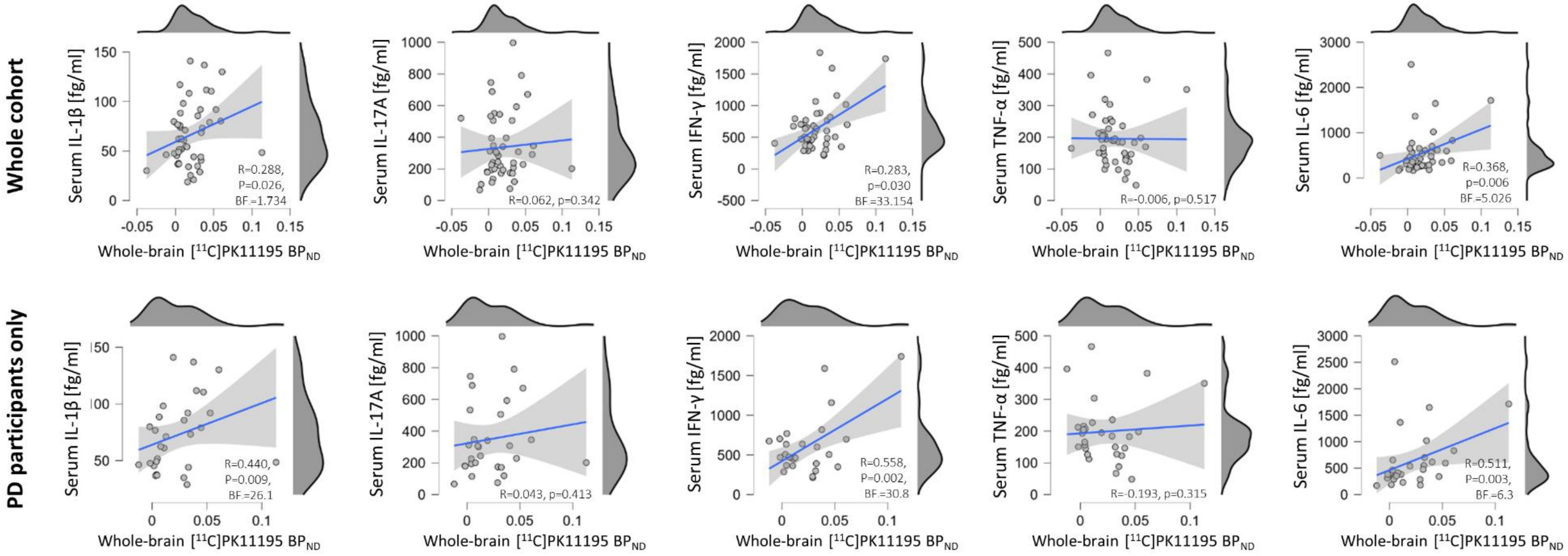

Supplementary Figure 2: Whole-brain grey matter [^{18}F] PK11195 BP$_{ND}$ correlates with three out of five serum cytokines. Serum cytokines were assessed using Meso Scale Discovery (Rockville) S-PLEX electrochemiluminescent immunoassays. All correlations that were significant at the whole population/cohort level remained significant when only assessing PD participants on their own. Grey shading is 95% confidence interval, with density plots for X and Y variables in dark grey.

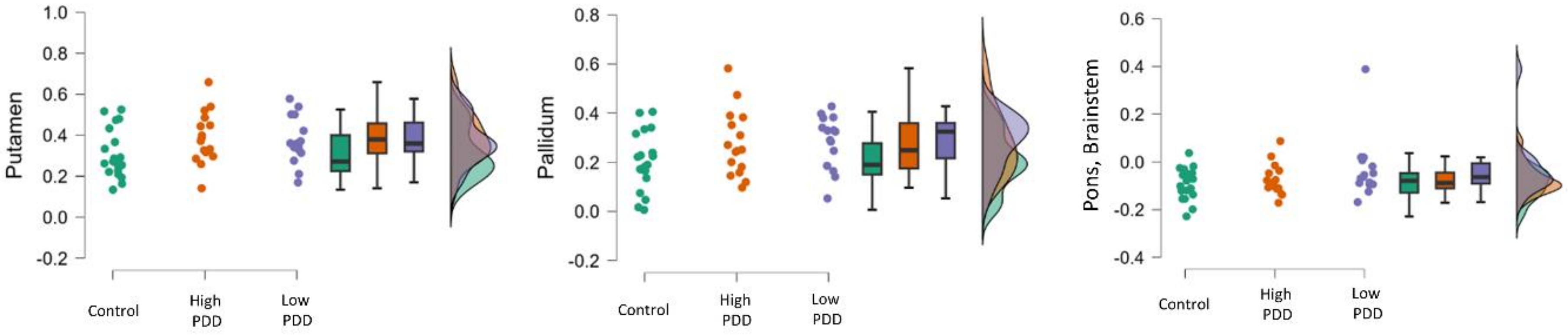

Supplementary Figure 3: Density raincloud plots of [^{18}F]AV-1451 BP_{ND} in regions that showed significantly greater binding in PD high (putamen) or low (pallidum, pons) dementia risk patients compared to controls. No significant differences between the dementia risk groups were observed. These regions are typically considered off-target binding regions for the AV-1451 ligand.
